# Implantable loop recorders in Brugada syndrome: Authors’ reply

**DOI:** 10.1093/europace/euac150

**Published:** 2022-09-03

**Authors:** Christopher Balfe, Rory Durand, Derek Crinion, Deirdre Ward, Richard Sheahan

**Affiliations:** Beaumont Hospital, Beaumont, County Dublin, Ireland; Cardiac Risk in Young Persons Clinic, Tallaght University Hospital, Tallaght, County Dublin, Ireland; Cardiac Risk in Young Persons Clinic, Tallaght University Hospital, Tallaght, County Dublin, Ireland; Cardiac Risk in Young Persons Clinic, Tallaght University Hospital, Tallaght, County Dublin, Ireland; Beaumont Hospital and Royal College of Surgeons, Beaumont, County Dublin, Ireland

We read with interest the recently published study by Scrocco *et al*.^1^ entitled ‘Role of subcutaneous implantable loop recorder for the diagnosis of arrhythmias in Brugada syndrome: a United Kingdom single-center experience’.

We commend Scrocco *et al*. on their excellent study, which includes the largest number of patients with implantable loop recorders (ILRs) implanted for Brugada syndrome to date.

A total of 50 patients with Brugada syndrome [deemed not to be at sufficient risk to mandate immediate implantable cardioverter defibrillator (ICD) implantation] received an ILR and were followed up for a median of 28 months. All except four of these patients were symptomatic with syncope, presyncope, or palpitations. The ILR monitoring identified actionable events (detection of an arrhythmia which led directly to a change of medical or device therapy) in 22% of these patients.

The authors provide a detailed breakdown of ILR findings according to the symptom which had triggered implantation. In patients with a recurrence of syncope or presyncope, an ILR-guided diagnosis was made in 57% (two patients had prolonged sinus pauses, one patient had paroxysmal complete atrioventricular block with pauses of up to 15 seconds, and one patient with paroxysmal AF had daytime pauses in the range of 2.2 to 6 seconds while on a low-dose beta-blocker).

In those patients with recurrent syncope or presyncope without actionable events on ILR monitoring, we note that the ILR was nevertheless helpful in facilitating the pursuit of an alternative cause, or in providing reassurance.

The authors also compare the characteristics and outcomes for patients who received an ICD for primary prevention, an ILR or no device therapy. Those patients who underwent implantation of a primary prevention ICD were more likely to have had a spontaneous Type 1 electrocardiogram pattern, had a broader QRS duration, and a higher prevalence of fragmented QRS, compared with those patients in the ILR group. Three patients in the ICD group (58 patients) received appropriate shocks for sustained ventricular tachycardia/ventricular fibrillation, while no life-threatening arrhythmias were detected in the patients who had received an ILR. This clinical outcome comparison from Scrocco *et al*. highlights the importance of a careful, multiparametric risk analysis prior to decision-making in patients with Brugada syndrome.

The median time from ILR implantation to the identification of actionable events was 19 months in this study, but with a range of up to 68 months. This suggests that there are a group of patients who will benefit from reimplantation of a second ILR for continued monitoring.

We congratulate Scrocco *et al*. on these important findings from their recently published manuscript and agree that this study would have added value to our publication on the evidence for the implantable loop recorder in patients with inherited arrhythmia syndromes.^2^ Unfortunately, as our literature review was submitted and accepted prior to the publication of this study, it was regrettably not possible to include these findings.^3^
